# Cytotoxic effects of persistent organic pollutants on the freshwater snail (*Lanistes carinatus*) in Kafr El-Zayat, Egypt

**DOI:** 10.1007/s10661-019-7751-2

**Published:** 2019-10-30

**Authors:** K. Y. Abdel-Halim, M. H. Mona, J. P. Giesy, F. A. Shouker, S. R. Osman

**Affiliations:** 1 Mammalian & Aquatic Toxicology Department, Central Agricultural Pesticides Laboratory, Agricultural Research Center (ARC), Dokki, 12618 Giza, Egypt; 2 Zoology Department, Faculty of Science, Tanta University, Tanta, Egypt; 3 Department of Veterinary Biomedical Sciences and Toxicology Centre, University of Saskatchewan, Saskatoon, Saskatchewan Canada

**Keywords:** Pollution, Mollusks, Monitoring, POPs, Oxidative stress

## Abstract

Effects of industrial and municipal wastewaters on the freshwater snail, *Lanistes carinatus*, were evaluated. Concentrations of some chemicals in some effluents were greater than permissible limits promulgated internationally by various jurisdictions. Pesticides and polychlorinated biphenyls (PCBs) observed in tissues of snails collected during summer were greater than those measured in snails collected during winter. Catalase activities observed during autumn were greater than those observed during other seasons. Activities of catalase were greater at all sites near sources of contamination than in snails from the reference site (S6). Lactate dehydrogenase activity was also greater at all sites relative to the location designated as the reference (S6), at which activities did not exceed 8.10 U/L. Patterns of genomic DNA in snails, as determined by use of OPA-02 primer, were significantly different among sites. Location S1 (Belshay village) exhibited 11 bands, followed by S2 (El-Demer zone) and S5 (Rosetta branch) which exhibited 6 bands. In contrast, all sites exhibited greater numbers of bands when the OPA-08 primer was used. Thus, DNA fingerprinting, lactate dehydrogenase, and catalase offer useful biomarkers in ecotoxicology and risk assessment programs.

## Introduction

Gastropods are ubiquitous in aquatic ecosystems and thus are prone to be exposed to various pollutants, including metals, industrial chemicals, pesticides, and hydrocarbons (Alonso and Camargo 2009; Hellou 2011). Snails have limited ability to metabolize xenobiotics (Moore and Livingstone 1987; Snyder 2000; Bonacci et al. 2007; Gagnaire et al. 2011; Hellou 2010). Exposure of snails to mixtures of chemical stressors can cause various effects in physiology and production of reactive oxidative stress. These effects have led to the development of biomarkers for rapid assessment of various types of contaminants to allow measures to be taken rapidly to minimize population or ecosystem-level effects and to develop appropriate restoration (Sarkar et al. 2006; Sarkar 2006). Uses of biomarkers at the molecular and cellular level are considered first-line sentinel tools that can be used as rapid, sensitive early warning measures of environmental quality (Torre et al. 2005). For example, the integrity of DNA can be affected by exposure to genotoxic agents that are clastogens that can cause breaks in strands of DNA, loss of methylation, and formation of adducts with DNA (Sarkar et al. 2006; Sarkar 2006). Studies of the integrity of DNA in the marine snail (*Planaxis sulcatus*) have shown effects of pollution at various locations, including breaks in strands of DNA. Lesser integrity of DNA was attributed to the extent of contamination by petroleum hydrocarbons in coastal waters (Sarkar et al. 2006). Alternatively, polychlorinated biphenyls (PCBs) are considered a ubiquitous contaminant due to releases from electrical transformers and hydraulics and other industrial uses. PCBs and their transformation products can cause the formation of reactive oxygen species (ROS) in the ambient environment causing oxidative stress that led to cell injury, mutagenesis, carcinogenesis, and cell death (Cerutti et al. 1989; Spitz et al. 2000). Random amplified polymorphic DNA polymerase chain reaction (RAPD-PCR) can be used to investigate the modification of the structure of DNA in living organisms (Conte et al. 1998). Moreover, a significant correlation was observed between intensities of gene flow of *Helicopsis striata* (Mollusca, Gastropoda, Plumonata) and geographical distances between populations in the South Central Russian Upland (Snegin 2017). There is a concern that isolated groups of this relict species could become extinct due to anthropogenic alterations of the environment, with the ongoing destruction of unique chalky biotopes, against the background of a significant decrease in allelic diversity (especially in industrial zones). Pesticides and PCBs are important groups of genotoxic environmental pollutants and their effects have been demonstrated by the use of in vivo and in vitro methods (Bolognesi 2003; Abdollahi et al. 2004; Kaushik and Kaushik 2007). Adaptive responses to exposure to contaminants meant to degrade or aid in depuration of contaminants include upregulation of mixed function monooxygenase (CYP) enzymes. The purpose of these enzymes is to insert oxygen into molecules to make them more polar, more easily conjugated, and more easily excreted and to make them more easily degraded. However, exposure to organic residues and the subsequent induction of electron-transport processes can result in the production of ROS that can result in oxidative damage, especially to membranes. The balance between benefits and adverse effects of antioxidant defenses (enzymatic and non-enzymatic) in a biological system can be used to integrate toxic effects under environmental conditions (Valavanidis et al. 2006). Random amplified polymorphic DNA (RAPD) was used with modification by using the polymerase chain reaction (PCR) to assess impacts of sub-lethal concentrations of two herbicides (glyphosate and atrazine) on the genotoxicity of *Biomphalaria glabrata* snails (Mona et al. 2013). Results of that study demonstrated that an amplified fragment of 500 bp could be generated by the use of OPA-10 primer to amplify DNA extracted from snails exposed to the herbicide. DNA (RAPD) was used to study the genetic structure of a population of the periwinkle *Littorina littorea* (Mollusca: Gastropoda) (Wolf et al. 2004). DNA was analyzed using RAPD to survey genetic structures of seven populations located along a gradient of contamination. Increases in expression of mRNA for lactate dehydrogenase (LDH) are a response to damage of tissues of snails caused by exposure to some xenobiotics (Bakry et al. 2011). Moreover, cumulative assessment of biomarkers in combination with measurement of concentrations of xenobiotics has the potential to monitor status and trends of aquatic environments of urban areas. Therefore, the present study was designed to assess effects of persistent organic pollutant (POPs) including selected pesticides and PCBs on genomic DNA fragmentation of the freshwater (Mollusca: *L. carinatus*) in the Kafr El-Zayat region, Egypt, that can be attributed to industrial and urbanized discharges.

## Materials and methods

### Study sites

**Fig. 1:**
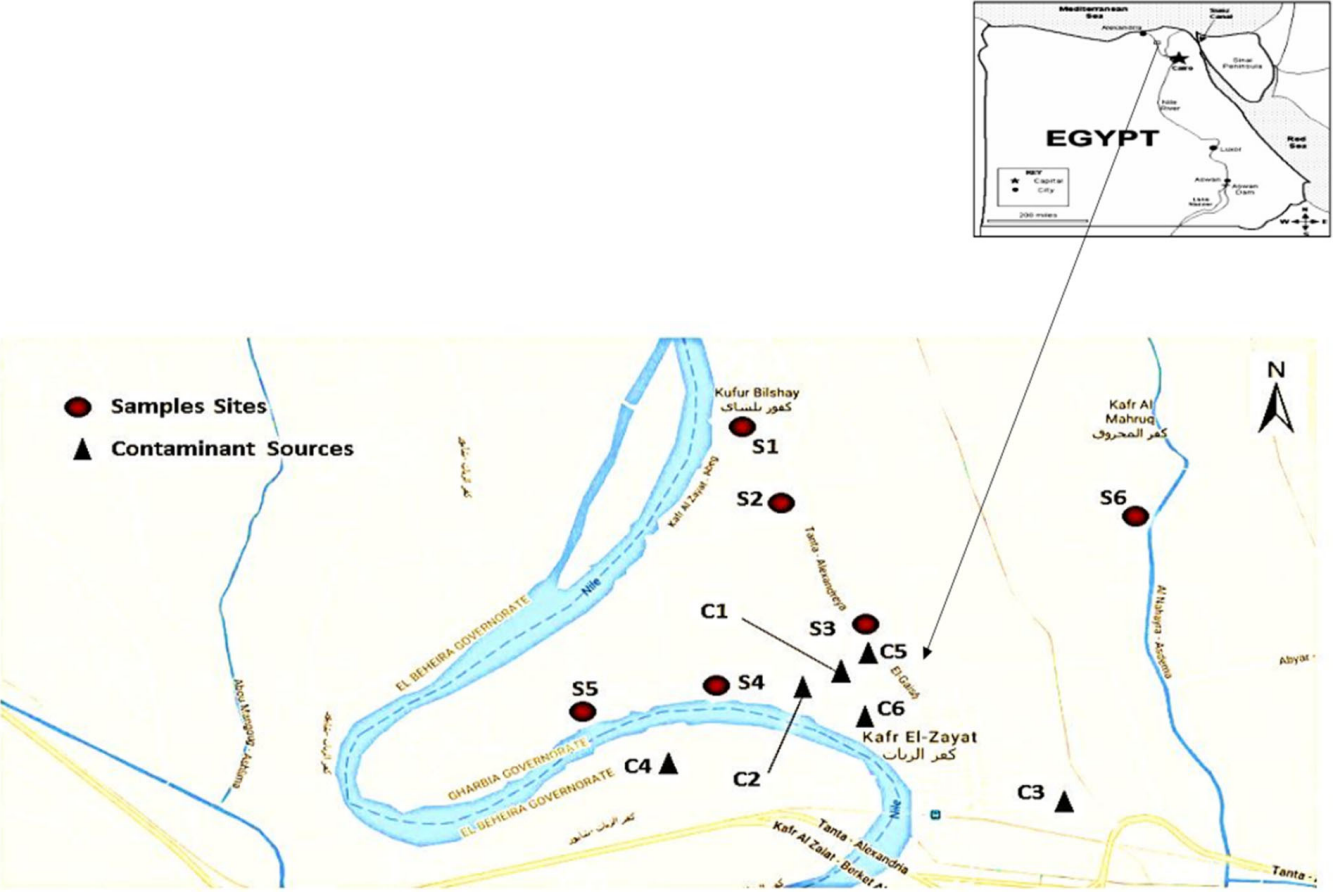
Google map showing potential sources of contamination and sampling sites of Kafr El-Zayat

**Fig. 2:**
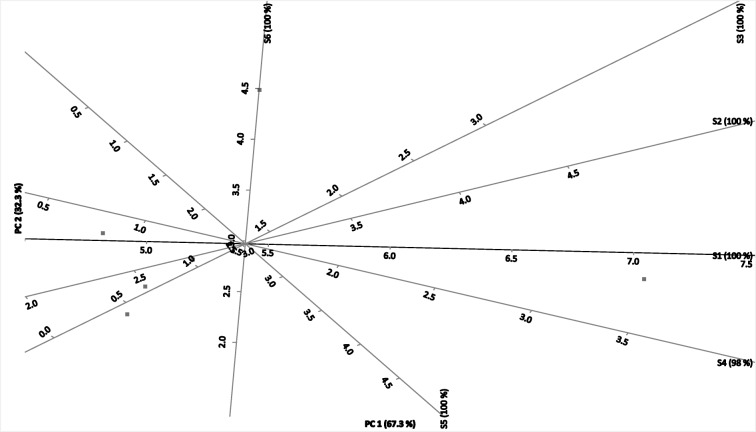
Correspondence analysis, triplot (Excel State program) of distance between contaminated sources (C1–C6) and selected sampling sites (S1–S6) of Kafr El-Zayat region, Egypt, represented correlations obtained after Pearson’s test (*P* < 0.05)

During this study, snails were collected from several sites in the Kafr El-Zayat district of Egypt, which lies on the Rosetta branch of the Nile River. This area was chosen because it is considered one of the main industrial regions in Egypt that includes a number of large factories, especially chemical manufacturers (IPEN 2006; Abdel-Halim et al. 2013). Five locations were selected near some of the major factories and urbanized areas for collection of snails. A more rural region was selected as a reference zone (S6) to compare (Fig. 1). These sites which were selected to cover various habitats of the Kafr El-Zayat freshwater environment (Fig. 2) were visited seasonally from March 2014 to February 2015.

### Collection of samples

Snails collected were mature with total body masses that ranged from 1–15 g. Mean size was approximately 25 mm (H) × 35 mm (D). Snails eat by grazing organic material that accumulates on plants, rocks, or soft bottom sediment. They scrape these surfaces using a unique life-like mouthpart called a radula (MELP 2000). Snails occur firmly attached to rocks, pieces of plastic, and rubber wheels, where they are usually present in colonies (EEAA 1999). Ten individuals of the snails *L. carinatus* with a total body weights ranging from 5 to15 g wet weight were collected by passing a dip net (30 cm × 40 cm) through the upper surface of sediments, water, and vegetation. Collected snails were kept in pre-labeled plastic containers and immediately transferred to the laboratory for further investigation. Hemolymph was collected by carefully inserting a 40-mm needle attached to a sterile 10-mL syringe under shells from the hemocoel along the right side of the head. All collected hemolymph was kept on ice during collection in sterile Eppendorf tubes − 20 °C until used. Consequently, snails were separated from their shells and dissected within 24 h of collection. Some organs such as the ganglia, digestive gland, and soft tissues were frozen at − 20 °C until used for quantification of pesticides, PCBs, or biomarkers. Samples of surface water (2 L) were collected in glass bottles, covered with screw caps lined with aluminum foil. Sediment was taken by using simplified equipment (auger) at a depth of 5 cm of sediment. The water was removed from the sediments before packed in a labeled polyethylene bag. The samples were air-dried in a dark place for 48 h before analysis.

### Analytes

Pure standards of *α*, *β*, and *γ* isomers of hexachlorobenzene (HCB), heptachlor (1-exo-hydroxychlordene), aldrin (1, 2, 3, 4, 10, 10*′*-hexachloro-1; 4, 4a, 5, 8, 8a-hexahydro-1; 4-endo-exo-5; 8-dimethanonaphthalene), heptachlor epoxide (1-exo-hydroxychlordene epoxide), dieldrin (hexachloroepoxy-octahydro-endo, exo-dimethanonahthalene), *γ*-chlordane (1, 2, 4, 5, 6, 7, 8, 8′-octachloro-3a, 4, 7, 7a-tetrahydro-4, 7-methanoindane), *P*,*P′*-DDE (*P*,*P′*-dichlorodihenyl dichloroethylene), endrin (hexachloro epoxy octahydro-endo, endo-dimethanonaphthalene), *P*,*P′*-DDD (2, 2 bis(*P*-chlorophenyl)-1, 1-dichloroethane), and *P*,*P′*-DDT (*P*,*P′*-dichlorodiphenyl trichloroethane) as well as a mixture of polychlorinated biphenyls (PCBs) consists of mono- to octachlorobiphenyls was obtained from EPA-Research Triangle Park, NC, USA. Multi-standards of insecticides, chlorpyrifos, chlorpyrifos-methyl, diazinon, pirimiphos-methyl, malathion, parathion, and profenofos, were supplied by Central Agricultural Pesticides Laboratory (CAPL), ARC, Egypt.

### Identification and quantification of pesticides

#### Snails

A quick, easy, cheap, effective, rugged, and safe (QuEChERS) technique was used to identify and quantify pesticides. Five grams of tissues was mixed with 10 mL of acidic acetonitrile (1% glacial acetic acid), and homogenized using a polytron homogenizer (Janke & KunKel, Gmb Hu Co KG) and shaken for 1 min. One gram of NaCl and 3 g of MgSO_4_ was added, vortexed immediately for 1 min, and centrifuged at 4000 rpm for 5 min. An aliquot (1 mL) was transferred into a micro-centrifuge tube (2 mL) containing 150 mg of primary secondary amine (PSA) and 200 mg MgSO_4_, vortexed for 1 min and re-centrifuged at 4000 rpm for 5 min. The supernatant was checked by the use of gas chromatography (Jiang et al. 2009).

#### Water

Samples of water were filtered through filter paper to remove sand, partitioned with 200 mL of methylene chloride, and concentrated to 1 mL. The extract was fractionated on deactivated silica gel (20%) column as described in the EPA protocol (USEPA 1982).

#### Sediment

Sieved, dried-sediments were mixed with acidic acetonitrile, MgSO_4_, NaCl, and sodium citrate. The mixture was then shaken for 30 s, sonicated for 5 min (ultrasonic bath at 50/60 Hz and 100 W, Barcelona, Spain) and centrifuged at 4000 rpm for 8 min. An aliquot of the supernatant was added to 1.5 g MgSO_4_ and 0.25 g PSA in 15-mL centrifuge tube. The solvent was evaporated to dryness, dissolved in 1 mL of cyclohexane, and filtered on polytetrafluoroethylene filters (Ramos et al. 2010).

Samples were analyzed by use of a model PAS-1701 gas chromatograph (Agilent, GC Model 6890) equipped with a fused silica capillary column of (30-m length × 0.32 mm i.d. and 0.25-μm film thickness). Oven temperatures were programmed from an initial temperature 160 °C for 1 min, ramped to 260 °C at a rate of 5 °C/min, and then held at 260 °C for 15 min. Nitrogen was used as the carrier gas at a flow rate of 3 mL/min. Residues were detected by use of an electron capture detector (ECD). Peaks identified and quantified by use of ECD were confirmed by use of mass spectrometry by use of an Agilent 6890 gas chromatograph equipped with Agilent mass spectrometric detector. Methods and instruments were fully validated as part of a laboratory quality assurances system (ISO/IEC 1990). The Codex Committee’s Criteria for quality assurance were followed to determine the accuracy and precision of the multi-residue method. Recovery was assessed by spiking 1 g of soft snail tissues with different levels of multi-standards of examined compounds and then compounds of interest were extracted and quantified as described above. Mean recoveries, expressed as percentages and limits of detection (LODs) for pesticides (organochlorine and organophosphate), and PCB compounds for fortified samples at several levels were determined and calculated for all tested compounds.

### Determination of acetyl cholinesterase activity in ganglial glands

Ganglial glands were weighed and homogenized in 20 volumes (w/v) of 0.1 M phosphate buffer, pH 8.0 (polytron homogenizer) for 15 s. Homogenates were centrifuged at 5000 rpm for 20 min with cooling (Sigma 3-30K5, Germany). Supernatants were used for quantification of enzyme activities by methods described previously (Ellman et al. 1961) with using acetylthiocholine iodide as the substrate. The activity of AChE was calculated as micromoles of substrate hydrolyzed per milligram of protein per min.

Activities were normalized to total protein quantified by the use of the method of Lowry et al. (1951). Bovine serum albumin (BSA) was used as a standard.

### Determination of catalase

The unit of CAT is the amount of enzyme which liberates half the peroxide oxygen from a solution of hydrogen peroxide of any concentration in 100 s at 25 °C. Catalase (CAT) activity was quantified by measurement of the decrease in absorbance at 240 nm due to the consumption of hydrogen peroxide (H_2_O_2_) (Beers and Sizer 1952). The reaction mixture consisted of 1 mL of 12.5 mM H_2_O_2_ (substrate), 2 mL of 66.7 mM phosphate buffer, pH 7.0, and an aliquot of enzyme source. The activity was expressed as unit per gram wet mass.

### Determination of lactate dehydrogenase

Lactate dehydrogenase activity of hemolymph was measured by the method of Mc Comb (1983). Hemolymph was prepared in 0.1 M phosphate buffer pH 7.5 in an ice bath. Sodium pyruvate was used as a standard and the enzyme activity was expressed as unit per liter.

### Fragmentation of DNA

#### DNA isolation

DNA was isolated from snail hemolymph by use of the solvent extraction procedure reported by Gupta (1996). Two hundred microliters of hemolymph was mixed with 200 μL of lysis buffer (LB) (200 mM Tris HCl pH 7.5 + 25 mM EDTA + 0.5% SDS). The mixture was incubated at 60 °C for 30 min with vigorously shaking. Samples were then centrifuged at 13.000 rpm for 5 min at room temperature. The supernatant was transferred into new tube, 5 μL of RNase A (10 mg/mL) added and incubated at 37 °C for 1 h. Proteinase K (50 μL/mL) was added and incubated overnight at 37 °C, followed by addition of 400-μL chloroform:phenol (1:1 v/v) mixed and centrifuged at 13.500 rpm for 10 min. The supernatant was transferred into a new tube, added 200 μL of isopropanol, mixed vigorously, and incubated at *−* 20 °C for 1 h. The supernatant was discarded and the pellets were washed with 400 μL of ethanol (70%), the tubes were centrifuged at 13.500 rpm for 10 min, the supernatant was discarded, and the pellets were dried at 50 °C for 15–30 min. They were resuspended in 80–100 μL of water-free nuclease.

#### Polymerase chain reaction

PCR master mix (2×) was suspended after thawing. Its composition for each sample was as follows: MgCl buffer (2.5 μL), primer (5.0 μL), dye DTNP (2.5 μL), template DNA (1.0 μL), Taq polymerase (0.2 μL), and water-free nuclease (14.0 μL). After addition, thermal cycling was done on the instrument (Aligent Technologies, Series Cycler 8800). The thermal cycling conditions were programmed as follows: initial denaturation was set at 95 °C for 30 min, denaturation at 95 °C for 30 s, annealing at 30 °C for 30 s, extension at 72 °C for 1 min, and ending to 72 °C for 5 min during 40 cycles.

Two primers were used as follows: OPA-02 (5′-TGCCGAGCTG-3′; G/C 70%) and OPA-08 (5′-AGCCACCGAA-3′; G/C 60%). Ten microliters of reaction yield and 2 μL (6×) loading dye were mixed, loaded on agarose gel, and examined against DNA maker or ladder for 20–30 min.

Gels were visualized and light scanned, and the data were analyzed on the basis of Jaccard´s coefficients to estimate the genetic similarities using the unweighed pair group method with arithmetic average (UPGMA) clustering algorithm to generate a dendrogram (Sneath and Sokal 1973).

### Statistical analyses

Analysis of variance (ANOVA) was used to compare means among treatments. Least square means were compared with significant differences between treatments using the Student-Newman-Keuls test (Sokel and Rohlf 1969). Multi-linear regressions between contaminated sites and biochemical variables were analyzed on the principal component analysis (PCA) (Hotelling 1933). The analysis was performed by use of Costat (Cohort software Inc 1985).

## Results

### Pesticides

Concentrations of measured compounds were adjusted for recovery (Table 1). During summer, concentrations of POPs in snails were greater than during winter. During summer, concentrations of pesticide residues were in decreasing order: S2 ˃ S3 ˃ S5 ˃ S1 ˃ S4 ˃ S6 (Table 2). The greatest concentration of 96 μg/kg wm was observed for ΣBHC, while the least concentration of 4.3 μg/kg wm was observed for heptachlor epoxide. Isomers of cyclodienes included heptachlor, aldrin, heptachlor epoxide, *γ*-chlordane, and endrin which had concentrations of 5.0, 38.0, 4.3, 5.9, and 10.0 μg/kg wm, respectively. Concentrations of ΣDDT did not exceed 54.0 μg/kg wm. Maximum concentrations of pesticides, diazinon and chlorpyrifos, were 8.2 and 11.0 μg/kg wm, respectively. In winter, the decreasing order was S5 ˃ S2 ˃ S1 ˃ S4 ˃ S3 ˃ S6. ΣBHC which had a maximum concentration of 68.0 μg/kg wm was the greatest concentration observed during winter, while the least concentration was 1.05 μg/kg wm for *α*-BHC. Concentrations of cyclodiene insecticides, heptachlor, endrin, and methoxychlor, were 3.8, 1.6, and 18.0 μg/kg wm, respectively, while the maximum concentration of ΣDDT was 26.0 μg/kg wm. Concentrations of diazinon and chlorpyrifos were 2.9 and 0.9 μg/kg wm, respectively.

**Table 1:** Recovery percentages and limit of detection (LOD) of measured pesticides and PCB congeners in various matrices

Compound	Fortified level (ng)	% of recovery			LOD (ng)
		Water	Sediment	Tissues	
HCB	2	56.4	48.19	95.0	0.003
*α*-BHC	2	31.7	74.12	98.0	0.002
*β*-BHC	16	84.1	57.53	87.8	0.005
*γ*-BHC	2	22.3	64.90	98.3	0.003
Heptachlor	2	100.2	55.86	99.0	0.004
Aldrin	2	100.5	87.22	93.7	0.003
Heptachlor epoxide	2	104.4	78.37	99.2	0.002
*γ*-Chlordane	4	62.8	64.36	87.7	0.005
Endrin	2	84.6	66.45	88.1	0.004
*P*,*P*′-DDE	2	92.3	59.51	99.0	0.002
*P*,*P*′-DDD	3	75.0	78.40	93.5	0.005
*P*,*P*′-DDT	4	105.3	92.00	93.4	0.006
Methoxychlor	4	73.6	95.60	90.2	0.005
Diazinon	38	72.2	110.30	99.8	0.050
Chlorpyrifos	60	95.0	72.57	90.4	0.070
ΣPCB_s_	2	70–110	78–113.0	87–99	0.003

*LOD*, limit of detection

**Table 2:** Concentrations of pesticides (ppb) in collected matrices from various zones in the Kafr El-Zayat district

Compound	Tissue									Water			Sediment		
	S1	S2	S3	S4	S5	S6	Range	Mean	C.V.%	Range	Mean	C.V.%	Range	Mean	C.V.%
Winter season															
*α*-BHC	BDL	BDL	BDL	BDL	6.31	BDL	BDL–6.31	1.05	0.39	BDL	BDL	–	BDL	BDL	–
*β*-BHC	BDL	BDL	BDL	BDL	354.22	BDL	BDL–354.22	59.04	3.08	8.79–185.86	56.22	1.46	BDL	BDL	–
*γ*-BHC	BDL	BDL	BDL	BDL	49.86	BDL	BDL–49.86	8.31	1.06	BDL–0.58	0.10	0.38	BDL	BDL	–
∑BHC	BDL	BDL	BDL	BDL	410.39	BDL	BDL–410.39	68.41	10.37	BDL–186.86	56.31	0.07	BDL	BDL	–
Heptachlor	BDL	BDL	BDL	BDL	19.32	BDL	BDL–19.32	3.81	0.14	BDL–14.22	4.82	0.27	BDL–0.80	0.13	0.08
Aldrin	BDL	BDL	BDL	BDL	BDL	BDL	BDL	BDL	BDL	BDL	BDL	–	BDL	BDL	–
Hept-epoxide	BDL	BDL	BDL	BDL	BDL	BDL	BDL	BDL	BDL	BDL–5.22	0.87	0.94	BDL–1.94	0.32	1.26
*γ*-Chlordane	BDL	BDL	BDL	BDL	BDL	BDL	BDL	BDL	BDL	BDL	BDL	–	BDL–4.85	0.81	0.51
*P*,*P*′-DDE	BDL	BDL	BDL	BDL	BDL	BDL	BDL	BDL	BDL	BDL–0.08	0.01	3.12	BDL–153.65	25.89	0.02
Endrin	BDL	BDL	BDL	BDL	9.60	BDL	BDL–9.60	1.60	0.25	BDL–7.66	2.08	0.67	BDL	BDL	–
*P*,*P*′-DDD	BDL	BDL	BDL	BDL	8.73	BDL	BDL–8.73	1.46	0.28	BDL–11.48	3.15	0.41	BDL–8.24	2.36	0.17
*P*,*P*′-DDT	BDL	BDL	BDL	BDL	147.72	BDL	BDL–147.72	24.62	3.12	BDL–0.24	0.04	1.44	BDL–57.44	13.69	0.04
∑DDT	BDL	BDL	BDL	BDL	156.45	BDL	BDL–156.45	26.08	1.02	BDL–11.80	3.21	0.14	BDL–153.65	41.93	0.01
Diazinon	BDL	BDL	BDL	11.48	5.66	BDL	BDL–11.48	2.86	0.18	BDL–40.65	12.27	0.04	BDL–18.24	3.04	0.13
Chlorpyrifos	BDL	BDL	BDL	BDL	5.29	BDL	BDL–5.29	0.88	0.46	BDL–37.65	9.61	0.06	BDL–3.01	0.52	0.81
Summer season															
*α*-BHC	9.40	13.48	8.87	8.88	12.71	BDL	BDL–13.48	8.89	0.05	BDL	BDL	–	2.30–210.39	82.76	0.06
*β*-BHC	77.14	53.87	75.53	64.87	89.04	BDL	BDL–89.04	60.08	0.01	BDL–3.62	0.94	0.54	BDL–2265.38	548.57	0.01
*γ*-BHC	13..03	36.52	88.34	11.75	11.44	BDL	BDL–88.34	26.85	0.02	BDL–157.56	28.09	0.09	BDL–4.73	0.79	0.52
∑BHC	99.57	103.87	172.74	85.50	113.20	BDL	BDL–172.74	95.81	0.14	BDL–157.56	14.52	0.55	BDL–2265.38	632.12	1.54
Heptachlor	3.91	4.83	8.64	BDL	7.67	4.78	BDL–8.64	4.97	0.08	BDL–0.42	0.10	5.27	BDL–278.73	47.52	0.01
Aldrin	12.59	83.46	123.86	BDL	BDL	7.34	BDL–123.86	37.88	0.02	BDL–0.87	0.16	2.51	218.26–9143.62	4451.91	0.01
Hept-epoxide	BDL	4.53	20.96	BDL	BDL	BDL	BDL–20.96	4.25	0.12	BDL–0.28	0.05	8.57	BDL–156.72	37.12	0.02
*γ*-Chlordane	6.41	6.89	3.49	BDL	18.63	BDL	BDL–18.63	5.90	0.11	BDL–0.27	0.06	8.97	BDL	BDL	–
*P*,*P′*-DDE	15.58	52.70	13.80	BDL	BDL	BDL	BDL–52.70	13.68	0.04	BDL	BDL	BDL	BDL	BDL	–
Endrin	BDL	28.80	20.86	BDL	11.14	BDL	BDL–28.80	10.13	0.05	5.51–35.64	12.97	0.04	BDL–245.78	70.14	0.01
*P*,*P′*-DDD	BDL	BDL	BDL	BDL	17.79	BDL	BDL–17.79	17.79	0.14	BDL–4.35	1.61	0.25	BDL–8.24	1.37	0.01
*P*,*P′*-DDT	21.33	34.36	20.93	35.50	54.53	57.14	20.93–57.14	37.30	0.32	BDL–0.28	0.16	6.19	BDL–225.28	41.66	0.01
∑DDT	36.91	87.06	34.73	35.50	72.32	57.14	34.73–87.06	53.94	0.09	BDL–4.35	0.89	0.48	BDL–225.28	43.03	0.01
Diazinon	BDL	BDL	18.82	17.03	13.08	BDL	BDL–18.82	8.16	0.07	BDL–246.23	74.53	0.01	BDL–42.50	11.88	0.05
Chlorpyrifos	14.78	9.60	10.12	13.41	14.60	BDL	BDL–14.78	10.42	1.42	14.10–305.47	91.29	0.05	BDL–7.37	1.66	0.31

*C.V.*, coefficient of variation; *BDL*, below detection limit. Sampling sites: S1 Belshay, S2 El-Demer, S3 Afifi, S4 Benoufer, S5 Nile River, and S6 rural zone (reference)

In water, mean concentrations of pesticides during winter were greater than during summer. During winter, the greatest concentration of 56.22 μg/L was observed for *β*-BHC, while the least concentration was 0.01 μg/L and was observed for *P*,*P′*–DDE. Isomers of cyclodiene insecticides, e.g., heptachlor, heptachlor epoxide, and endrin exhibited 4.82, 0.87, and 2.08 μg/L, respectively, while aldrin and *γ*-chlordane were below their detection limits. Concentrations of ∑DDT exhibited 3.21 μg/L. Diazinon and chlorpyrifos exhibited 12.27 and 9.61 μg/L, respectively. During summer, the greatest concentration of 91.29 μg/L was observed for chlorpyrifos followed by 74.53 μg/L which was observed for diazinon. *γ*-BHC exhibited a mean concentration of 28.09 μg/L, while heptachlor epoxide exhibited the least concentration of 0.05 μg/L. Other cyclodiene insecticides, heptachlor, aldrin, *γ*-chlordane, and endrin, were 0.10, 0.16, 0.06, and 12.97 μg/L, respectively. The concentrations of ∑DDT were 0.89 μg/L.

Concentrations of pesticides in sediments during summer were greater than those during winter. During summer, the greatest mean concentration 4451.91 μg/kg dry mass (dm) was observed for aldrin, followed by 632.12, 548.5, 82.76, and 70.14 μg/kg dm for ∑BHC, *β*-BHC, *α*-BHC, and endrin, respectively. *γ*-BHC exhibited the least mean concentration of 0.79 μg/kg dm, while *γ*-chlordane and *P*,*P′*-DDE were below their detection limits. ∑DDT exhibited a mean concentration of 43.03 μg/kg dm. Concentrations of diazinon and chlorpyrifos were 11.88 and 1.66 μg/kg dm. During winter, the greatest mean concentration of 41.93 μg/kg dm was observed for ∑DDT, while the least mean concentration of 0.13 μg/kg dm was observed for heptachlor. DDT isomers exhibited mean concentrations of 25.89, 2.36, and 13.69 μg/kg dm for *P*,*P′*-DDE, *P*,*P′*-DDD, and *P*,*P′*-DDT, respectively. Other cyclodiene insecticides did not exceed 0.81 μg/kg dm which was observed for *γ*-chlordane. Concentrations of diazinon and chlorpyrifos were 3.04 and 0.52 μg/kg dm, respectively.

### PCBs

During summer, when the mean concentration was 7.4 μg/kg wm, concentrations of PCB congeners were greater than those observed in snail tissues during winter (Table 3). The greatest concentration of an individual PCB congener was 2.0 μg/kg wm, which was observed for congener 52, while the congener 189 exhibited the least concentration among congeners of 0.2 μg/kg wm. During winter, the greatest concentration of a congener was 5.5 μg/kg wm for congener 52, while the least concentration was 0.01 μg/kg wm for congeners 99, 140, and 128, respectively. In water, concentrations of ∑PCBs congeners during summer were greater than during winter. During summer, the greatest mean concentration of an individual PCB congener was 8.58 μg/L, which was observed for congener 52, while the congener 70 exhibited the least concentration among congeners of 0.11 μg/L. During winter, ∑PCBs did not exceed 2.79 μg/L. The greatest concentration of 1.73 μg/L was observed for congener 52, while the least concentration of 0.02 μg/L was observed for congener 128.

**Table 3:** Concentrations of polychlorinated biphenyls (PCBs) (ppb) in collected matrices from various zones in the Kafr El-Zayat district

PCBs	Tissue									Water			Sediment		
	S1	S2	S3	S4	S5	S6	Range	Mean	C.V.%	Range	Mean	C.V.%	Range	Mean	C.V.%
Winter season															
18	BDL	BDL	BDL	BDL	1.53	BDL	BDL–1.53	0.26	1.60	BDL–0.69	0.12	0.36	BDL–0.19	0.03	1.33
52	0.06	0.09	0.07	BDL	2.88	BDL	BDL–2.88	0.52	1.03	BDL–6.22	1.73	0.03	BDL–4.65	0.89	0.58
31	BDL	BDL	BDL	BDL	BDL	BDL	BDL	BDL	BDL	BDL	BDL	–	BDL	BDL	–
70	BDL	BDL	BDL	BDL	BDL	BDL	BDL	BDL	BDL	BDL	BDL	–	BDL	BDL	–
101	BDL	BDL	BDL	BDL	1.30	BDL	BDL–1.30	0.22	1.88	BDL	BDL	–	BDL–0.69	0.13	4.32
140	BDL	0.05	BDL	BDL	BDL	BDL	BDL–0.05	0.01	0.49	BDL–0.73	0.12	0.34	BDL–0.03	0.02	4.22
105	BDL	BDL	BDL	BDL	0.40	BDL	BDL–0.40	0.07	6.12	BDL	BDL	–	BDL–0.003	0.001	1.54
138	BDL	BDL	BDL	BDL	2.32	BDL	BDL–2.32	0.39	1.05	BDL	BDL	–	BDL–0.90	0.17	4.25
128	BDL	0.03	BDL	BDL	BDL	BDL	BDL–0.03	0.01	0.82	BDL–0.11	0.02	3.54	BDL–0.19	0.03	1.31
189	0.04	0.11	0.07	BDL	0.34	BDL	BDL–0.34	0.09	0.53	BDL–2.72	0.81	0.07	BDL–0.15	0.07	1.18
∑PCB_s_	0.10	0.28	0.14	BDL	8.77	BDL	BDL–8.77	0.16	1.58	BDL–6.22	2.79	0.28	BDL–4.65	1.34	0.38
Summer season															
18	1.44	2.42	3.94	1.78	2.35	BDL	BDL–3.94	1.99	0.21	BDL–6.70	1.12	3.65	BDL–22.87	4.39	0.12
52	BDL	0.81	0.75	BDL	BDL	4.62	BDL–4.62	1.03	0.53	4.66–15.23	8.58	0.44	BDL–17.92	4.88	0.11
31	0.72	1.54	1.62	0.43	BDL	1.07	BDL–1.62	0.90	0.45	BDL–5.14	2.16	1.98	1.19–519.54	238.20	0.01
70	BDL	0.88	BDL	BDL	BDL	0.90	BDL–0.90	0.30	1.74	BDL–0.67	0.11	3.62	BDL–24.71	5.89	0.10
101	3.70	1.11	3.61	1.88	BDL	BDL	BDL–3.70	1.72	0.30	BDL	BDL	–	BDL–0.80	0.13	3.06
101	3.70	1.11	3.61	1.88	BDL	BDL	BDL–3.70	1.72	0.30	BDL	BDL	–	BDL–0.80	0.13	3.06
140	BDL	0.62	0.62	BDL	BDL	BDL	BDL–0.62	0.21	0.02	BDL–0.84	0.14	2.88	BDL–3.66	0.97	0.53
105	0.72	1.04+	0.75	BDL	BDL	BDL	BDL–1.04	0.42	0.18	BDL–0.5	0.12	4.82	BDL–8.99	1.50	0.27
138	BDL	0.103	0.41	0.56	BDL	0.85	BDL–0.85	0.32	0.18	BDL–1.35	0.44	1.06	BDL–3.54	0.64	0.80
128	BDL	0.56	0.54	BDL	BDL	BDL	BDL–0.56	0.18	0.03	BDL–0.22	0.06	8.31	BDL–5.88	0.98	0.42
189	BDL	0.60	BDL	BDL	BDL	0.32	BDL–0.60	0.15	0.04	0.92–20.80	6.94	0.92	BDL–0.33	0.10	5.43
∑PCB_s_	6.59	10.68	12.22	4.64	2.35	7.77	2.35–12.22	7.38	1.58	BDL–34.76	20.55	1.04	BDL–519.5	259.9	0.01

*C.V.*, coefficient of variation; *BDL*, below detection limit. Sampling sites: S1 Belshay, S2 El-Demer, S3 Afifi, S4 Benoufer, S5 Nile River, and S6 rural zone (reference)

Concentrations of PCBs congeners in sediments during summer were greater than those during winter, where ∑PCBs exhibited 259.9 μg/kg dm during summer. The greatest concentration of an individual PCB congener was 238.20 μg/kg dm, which was observed for congener 31, while the congener 189 exhibited the least concentration among congeners of 0.01 μg/kg dm. During winter, ∑PCBs did not exceed 1.34 μg/kg dm. The greatest concentration of 0.89 μg/kg dm was observed for congener 52, while the least concentration of 0.001 μg/kg dm was observed for congener 105.

### Biochemical responses

#### Acetyl cholinesterase activity

**Fig. 3:**
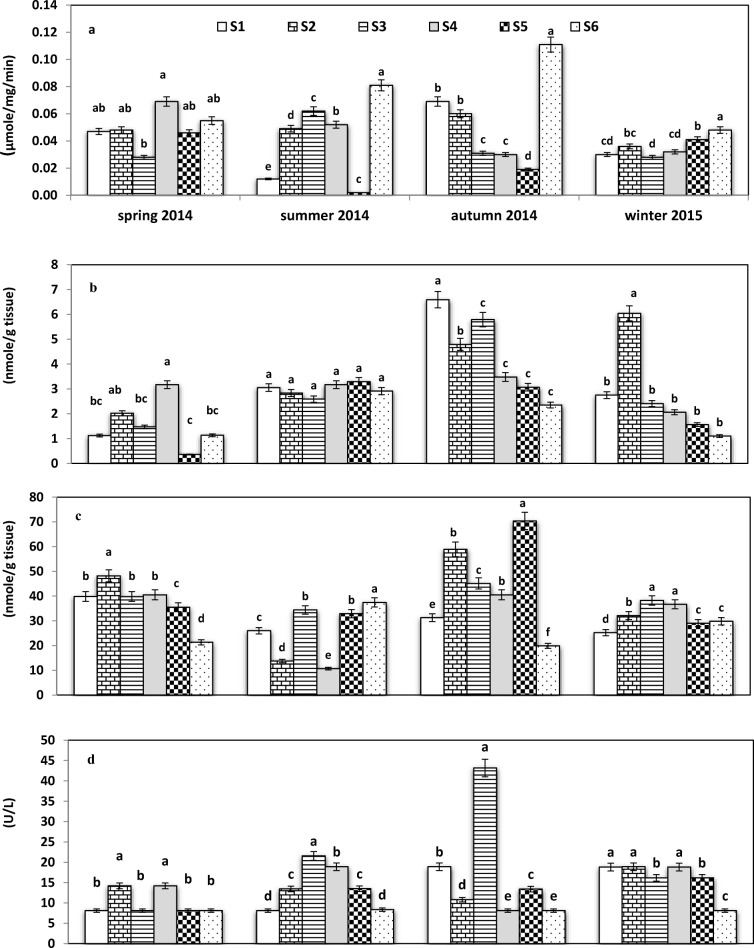
Activities of **a** AChE (μmol/mg/min) in ganglial gland, **b** CAT in whole body, **c** CAT in hemolymph (mmol/g tissue), and **d** LDH hemolymph (U/L), respectively, of the freshwater snail, *L. carinatus* from Kafr El-Zayat region. Vertical bars indicate standard errors. Means with the same letters are not significantly different (*P* < 0.05)

Activities of AChE in ganglial glands of freshwater snails *L. carinatus* during various seasons were as follows: autumn > spring > summer > winter, respectively (Fig. 3a). During autumn, activities at each location, including S6, which was selected as the reference location, decreased in the order as follows: S6 > S1 > S2 > S3 > S4 > S5, with mean values of 0.11, 0.07, 0.06, 0.03, 0.03, and 0.02 μmol/min/mg protein, respectively. Activities of AChE in snails from S6 (rural site) were greater than those in snails from other sites during all seasons, except during spring. Site 5 exhibited the greatest proportion of individuals exhibiting inhibition as a percentage of collected animals (2.79 and 17.3%) during summer and autumn, respectively. During spring, there was no statistically significant difference between mean activities at S1 and S2, but S3 exhibited the least activity.

#### Catalase

Mean activities of CAT in the tissues of *L. carinatus* collected during autumn were greater than that during other seasons (Fig. 3b). During this period, CAT activities in snails from various locations were in decreasing order as follows: S1 > S3 > S2 > S4 > S5 > S6, with mean activities of 6.59, 5.49, 4.79, 3.48, 3.07, and 2.35 nmol/g tissue. In hemolymph, CAT activity during autumn was greater than during other seasons and was in the decreasing order as follows: S5 > S2 > S3 > S4 > S1 > S6 with mean activities of 70.0, 59.0, 45.0, 41.0, 31.3 and 20.0 nmol/g tissue (Fig. 3c). In fact, S6 (rural site) recorded the lowest values in whole-body tissue during autumn and winter seasons, while during spring and autumn in the hemolymph.

#### Lactate dehydrogenase

Activities of LDH in hemolymph were greater at all locations relative to those in snails from the reference site (S6), which did not exceed 8.10 U/L (Fig. 3d). LDH activities varied seasonally with mean season activities decreasing in the order: autumn > winter > summer > spring. During winter, there were no significant different differences LDH activities in snails collected from any of the sites compared with that in snails from the reference site. Among the more impacted sites, S3 imposed the highest contaminated zone followed by S4 and S3. The greatest LDH activity was observed in the hemolymph of snails from S3, which was 43.17 U/L during autumn, while the least activity was observed in snails from S1, where the mean activity was 8.10 U/L during spring and summer.

#### Correlation analysis

**Fig. 4:**
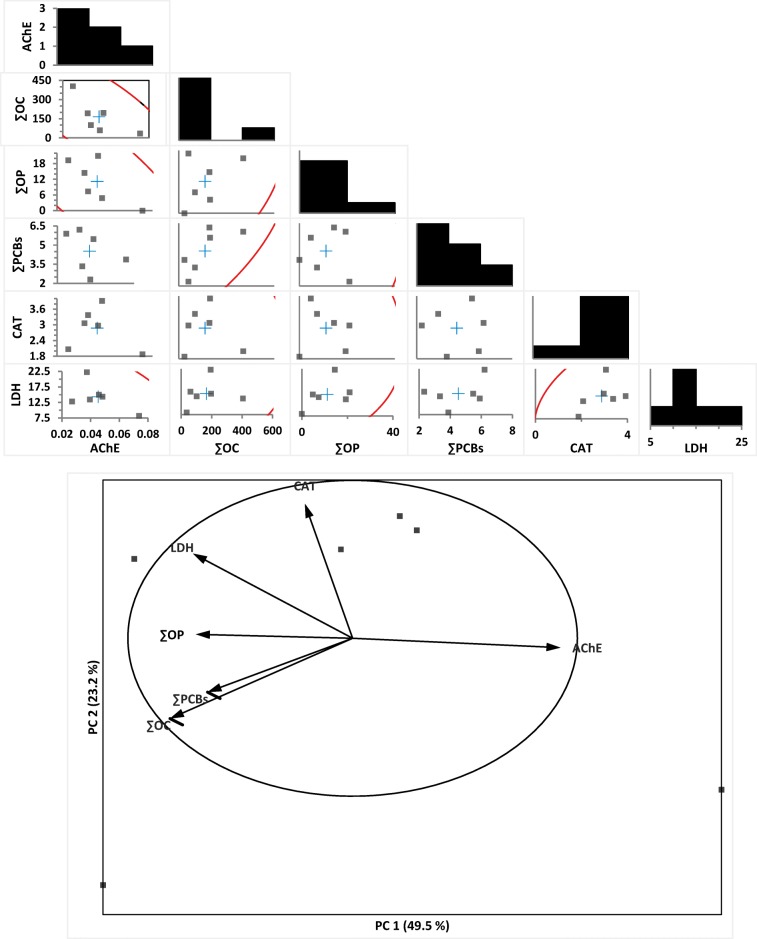
Correlation matrix of concentrations POPs in tissues of the freshwater snail; *L. carinatus* and biochemical variables: **a** column diagram represents the association between variables AChE, CAT, and LDH with concentrations of OCs, OPBs in snails (*N* = 6 sites); linear correlation between these variables with described POPs residue levels obtained after Pearson’s test (*P* < 0.05)

There were significant (*P* ˂ 0.05), negative correlations between AChE and OCs, OPs, and PCBs, with Pearson correlation coefficients of − 0.74*, − 0.73*, and − 0.389, respectively (Fig. 4). However, a slight negative correlation between activities of CAT and concentrations of both OCs and OPs (*r* = − 0.128 and − 0.072). Activities of LDH were positively associated with concentrations of OCs, OPs, and PCBs (*r* = 0.207, 0.481, and 0.410, respectively).

#### Genomic DNA diversity

**Fig. 5:**
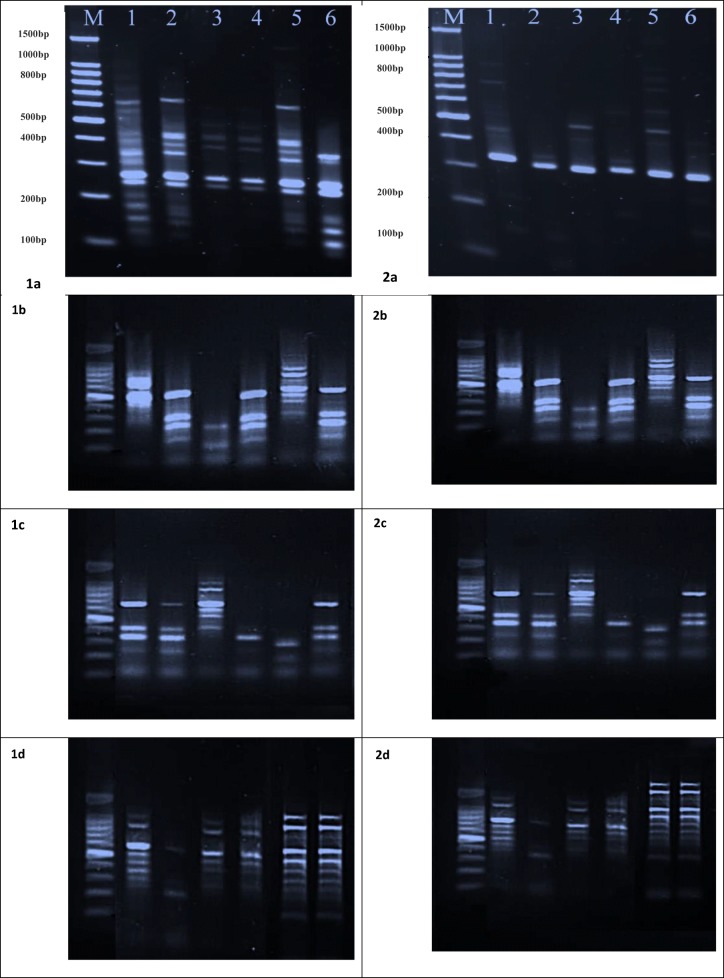
Agarose gel electrophoresis of polychain reaction (RAPD-PCR) products using (column 1) primer; OPA-02 and (column 2) primer; OPA-08 to amplify *L. carinatus*: Lan (1) DNA ladder and samples from S1 to S6 during: **a** spring, **b** autumn, **c** summer, and **d** winter

When genomic DNA of *L. carinatus* collected from six sites of Kafr El-Zayat district was amplified using RAPD-PCR with primers OPA-02 and OPA-08 (Fig. 5), different patterns were observed. This variation among genetic diversities among *L. carinatus* was stated by using similarities coefficients and dendrogram tree. The two primers applied successfully amplified genomic DNA from snails from each site. Amplifications using the two primers produced RAPD fingerprints with varying numbers of bands ranging from 100 to 1000 bp depending on the status of exposed animals and the primer used.

There are significant differences patterns of genomic DNA, based on patterns in agarose gels. The number of unique bands of DNA oligomers amplified and represented by individual, discernible bands that ranged in size from 200 to 600 bp. The number of bands generated by the use of primer OPA-02 resulted in more bands than did primer OPA-08, especially during spring. When snails from all sites exhibited more amplified and polymorphic bands generated by the use of the OPA-02 primer than with the OPA-08 primer. Total numbers of bands ranging in size from 200 to 600 bp amplified using OPA-02, for snails from sites 1, 2, and 5 were 11. In contrast, sites 3 and 4 exhibited the fewest bands. When OPA-08 primer was used as the primer in RAPD analyses, all sites exhibited fewer bands, the number of which ranged from 1 to 3. The estimated similarity coefficient among groups of snails ranged from 20 to 80% during spring. The greatest values (80%) were observed for snails from locations S1 and S2 and between S5 and S6, while the least similarity of 60% was observed for locations S3 and S4 when primer OPA-02 was used as the primer.

During autumn and summer, no significant differences were observed in the number of bands produced by the use of the two primers. When primer OPA-02 was used, similarity coefficients among groups were least (50%) between S1 and S5, but greatest (80%) between S2 and S4. In case of primer OPA-08, the least similar sites (50%) were S1 and S5 followed by sites 2, 4, and 6, while the greatest similarity (80%) was observed between S1 and S5, S2 and S4, and S3 and S6. During winter, primer OPA-02 generated the greatest number of bands ranged from 200 to 600 bp for snails from locations 1, 5, and 6, while the fewest bands were observed for snails from S2 followed by those from sites S3 and S4. Total numbers of bands amplified with primer OPA-02, at sites S5 and S6, were 9 bands, followed by S1 with 8. Snails from both S3 and S4 exhibited 6 bands. Snails from site 2 only exhibited bands of less than 200 bp. When OPA-08 primer was used as the snails from all sites exhibited 1 to 3 bands and the fewest bands were observed for snails from sites 3 and 4 exhibited only two bands during spring. Similarity coefficients were least (50%) for snails from sites S5 and S6, and greatest for sites S3 and S4. A moderate similarity (62%) was observed between snails from either sites 1 and 2 or between 3 and 4 during most seasons.

## Discussion

The present study focused on distributions of chemical pollutants, in surface waters of the Kafr El-Zayat district and their effects on several biomarkers of the health of the freshwater snail. Concentrations of contaminants varied among seasons in association with industrial effluent discharges of local surface waters and runoff urbanized regions. The fact that mean concentrations of persistent organic pollutants (POPs), e.g., organochlorine pesticides (OCPs) were found during winter and greater concentrations were observed during summer might be due to effects of precipitation of discharge, pH, temperature, microbial status, and mineralization.

The presence of various factors could have resulted in an environmental condition which facilitated dilution, dissipation, and possibly for some compounds transformation or the degradation in aquatic media. The slightly alkaline water might have enhanced dehydrochlorination of DDT to its metabolites, DDE and DDD (Abu El-Amayem et al. 1979). In addition, minerals and salts can promote photodecomposition, especially during intense solar radiation such as is observed in Egypt (Schlauch 1989). Discharges from both industry and domestic wastewaters also contained small organic molecules (sensitizers) that can act as catalysts of photo-degradation directly or indirectly by transfer of electrons (Acher and Rosenthale 1977; Bowman and Sans 1980; Schlauch 1989; Larson et al. 1989; Lymann et al. 1990). During summer, concentrations of PCBs were greater than in winter at all locations. This might be due to greater activities of industries and in urban areas, especially at factories making brick near sites S3 and S5. However, in tissues of some gastropod, diazinon and chlorpyrifos were the only residues accumulated. This fact explains the role of physicochemical properties of either diazinon or chlorpyrifos in their persistent in tissues or organic matter of sediment depending on heterocyclic contains (pyridine rings). Compounds detected in surface water were consistent with the fact that pesticides and fertilizers are the main products of the Kafr El-Zayat Company, and consistent with results of previous studies (Dogheim et al. 1996; IPEN 2006; Abdel-Halim et al. 2013; Abdel-Halim 2014). Those studies documented that organochlorine, organophosphorus, pyrethroid pesticides, PCBs, PAHs, and heavy metals were detected as by-products of industrial and urbanized activities in the Kafr El-Zayat region. Concentrations of the pesticides DDT as well as products of transformation, DDD, DDE, and profenofos and diazinon were the most detected in food and environmental samples.

Reduction in AChE observed during this study might be attributed to exposure of snails to anticholinesterase compounds present at these sites. The greatest inhibition was observed during summer and autumn, especially in snails from sites 4 and 5 near the discharges coming from either Kafr El-Zayat of pesticides or El-Malyia Company for fertilizers. In comparative with the control zone, S6 exhibited great activities of AChE in collected animals with those collected from more contaminated sites. Cholinesterase was considered to be a good diagnostic tool for chronic exposure to organophosphate (OPs) and carbamate insecticides (Dembele et al. 2000; Bakry et al. 2001). Various factors can affect concentrations of organophosphates in drainage water such as presence of most minerals and salts (Schlauch 1989) as well as numbers of microorganisms (Haven and Rase 1990). Use of AChE as a biomarker for assessment of ecotoxicological effects on freshwater snails is limited (Laguerre et al. 2009). Previously, some investigators have used it as a specific biomarker to assess the exposure of aquatic organisms to OPs and carbamates in laboratory and field studies (Bocquené et al. 1997; Scops et al. 1997; Galloway et al. 2002; Binelli et al. 2006) and also to other contaminants such as metals, synthetic detergents, some components of fuel, oils, and algal toxins (Payne et al. 1996; Guilhermino et al. 1998; Lehtonen et al. 2003).

Hemolymph of snails has been used to assess activities of both CAT and LDH and then correlated with genotoxic effects of contaminants that depended on production of ROS which affected various cellular processes, such as functions of membranes (Pinto et al. 2003; Valko et al. 2005). Potential of ROS to damage tissues and cellular components is called oxidative stress. ROS is also capable of interacting with DNA to form DNA adducts, which then leads to strand breaks of DNA. Possible anthropogenic-related sources of enhanced ROS and other pro-oxidant-free radical production include organic contaminants such as nitro aromatics, PAHs, PCBs, pesticides, and heavy metals (Lemaire and Livingstone 1993; Di Giulio et al. 1995; Halliwell and Gutteridge 1999). Among effects of pollutants discharged to surface waters, snails near S4 (near fertilizers factories) exhibited the greatest amount of oxidative stress, followed by S5 and S3, respectively. Previous studies demonstrated the oxidative stress in mollusks was adversely affected by various pollutants in coastal areas, marine, and freshwater. For example, in mussels, *Mytilus galloprovincialis* (Vlahogianni et al. 2007), *Perna perna* (Almeida et al. 2007), *Ruditapes philippinarum* (Sacchi et al. 2013), and freshwater mussel (Falfushynska et al. 2014). Moreover, Al-Fanharawi et al. (2019) conducted that significant alterations in CAT, AChE, superoxide dismutase (SOD), and DNA damage were induced in soft tissues of freshwater mussels *Unio tigridis* and snail *Viviparous benglensis* after exposure to chlorpyrifos for 21 days. Similar investigation was demonstrated by Banaee et al. (2019) on freshwater snail, *Galba truncatula* exposed to insecticide, dimethoate, for 14 days under laboratory conditions. Dimethoate induced oxidative stress and altered some biochemical parameters in tested animals. Another investigation stated that azinphos-methyl at concentrations of 20 and 200 μg/L for 14-day exposure induced decrease in cholinesterase and carboxylesterase activities in freshwater gastropod, *B. stramina*. Activities of CAT and glutathione-*S*-transferase (GST) and total antioxidant capacity (TAC) were significantly increased compared with untreated animals (Cossi et al. 2018).

Greater releases of LDH into hemolymph of snails from locations other than the reference area are indicative of cellular and membrane damage (Osman 1999; Bakry et al. 2011). Previously, greater activities of LDH have been observed in selected gastropods in polluted sites than in those from less contaminated reference sites. This result indicated that some oxidative injury might be related to exposure to POPs. Results of the present study that demonstrated a strong correlation between biomarkers freshwater snail, *L. carinatus*, and concentrations of several POPs were consistent with results of previous studies in other polluted regions which have demonstrated greater lipid peroxidation and expression of CAT in digestive glands of mussels *M. yunnanensis* (Torres et al. 2002) and snails; *Theba pisana* (El-Gendy et al. 2009; Radwan et al. 2010) and *Helix aspersa* (Abdel-Halim et al. 2013) compared with those in less contaminated reference areas.

Genomic DNA profile of snails collected seasonally from appropriate habitats of the Kafr El-Zayat district exhibited significant differences between and among sites. The seasonal variation among genomic DNA diversity is similar to those obtained in biomarkers of animals near emission. In general, the contents of industrial effluents not only disrupted the integrity of the genome but also affected the expression of DNA directly or indirectly (Shugart and Theodorakis 1996). These effects will lead to an increase in the incidence of different types of gene mutations and in the long-term result in the genetic variability of the exposed populations. In this context, random amplified polymorphism DNA (RAPD) is a powerful technique that involves the amplification of random segments of genomic DNA using PCR.

It has been previously reported that various POPs and metals can cause oxidative stress resulting in damaged DNA by generation ROS. Damage to DNA caused by ROS includes strand breaks, base modification, and basic sites (Livingstone 2000). In addition to attack by ROS, DNA strand breaks and mismatch errors also occurred due to incomplete excision repair of DNA adducts (Speit and Hartmann 1995). Changes in DNA fingerprints (e.g., DNA fragmentation) observed reflect DNA alterations in genome ranging from single base changes (point mutations) to complex chromosomal rearrangements (Atienzar et al. 1999 and 2000). Thus, DNA fingerprinting offers useful biomarker assay in ecotoxicology (Savya 1998). In this work, results of RAPD assay showed significant differences in RAPD patterns in polluted exposed groups compared with the reference group (control: non-exposed), with respect to variation in intensities of bands, the disappearance of bands, and appearance of a new band of amplified DNA.

## Conclusion

The use of a wide pattern of biomarkers allowed detecting biological and potential harmful effects of POPs into freshwater snails. In this work, AChE, antioxidant defense enzymes, LDH, and genotoxic effect (DNA damage) in digestive gland and hemolymph, respectively, were expected to inform on cell damage in environmentally exposed organism. These findings may state-imposed risk from industrial effluents and urbanized activity. These outcomes investigate good information among the ecosystem status of Kafr El-Zayat region and impose predicted risk of chemicals emitted from industrial units on aquatic organisms. Moreover, governmental and management decisions and control options must be done for environmental remediation resulting in good quality of ecosystem arising human health protection outcome.
